# Draft Genome Sequence of an *Enterobacter* Species Associated with Illnesses and Powdered Infant Formula

**DOI:** 10.1128/genomeA.01479-15

**Published:** 2016-01-14

**Authors:** Emily E. Jackson, Pauline Ogrodzki, Ben Pascoe, Samuel K. Sheppard, Stephen J. Forsythe

**Affiliations:** a Pathogen Research Group, School of Science and Technology, Nottingham Trent University, Nottingham, United Kingdom; b Institute of Life Science, College of Medicine, Swansea University, Swansea, United Kingdom

## Abstract

This is the first report of the draft genome sequence of an *Enterobacter* species that may have been transmitted from powdered infant formula (PIF) to infants, resulting in illness. *Enterobacter* spp. are currently permitted in PIF, but the transmission of this strain indicates that the microbiological criteria for PIF may need revision.

## GENOME ANNOUNCEMENT

In 2010, a hospital outbreak investigation attributed two cases of infant bloody diarrhea to *Cronobacter sakazakii* (1). Later characterization using multilocus sequence typing identified the patient isolates as *Enterobacter hormaechei* and an unidentified species of *Enterobacter* (2). *Enterobacter* species strains, including 8706, belonging to the same pulsotype were isolated from powdered infant formula (PIF), rehydrated PIF, the hospital environment, and both patients (1, 2). The presence of this pulsotype in both the PIF and infants suggests a possible common source, and this outbreak may be the first reported transmission of an *Enterobacter* species to infants from PIF (2). Currently, PIF is required to be microbiologically tested for *Cronobacter* and *Salmonella* only (3). Therefore, the genome sequencing of this *Enterobacter* species is warranted, as it is permitted in PIF but may present a risk to infants.

Bacterial DNA from strain 8706 was extracted from 1-day cultures using a GenElute bacterial genome kit (Sigma-Aldrich) and sequenced using the Illumina MiSeq sequencing platform. A total of 450,563 high-quality paired-end reads were generated with 60.1-fold coverage. *De novo* assembly was performed using SPAdes, with quality assessment using QUAST (4, 5). The genome consisted of 187 contigs, with a total size of 4,999,669 bp and a G+C content of 54.73%.

Phylogenetic trees based on six housekeeping genes (2,673 bp) and 53 ribosomal genes (22,511 bp) were generated from sequences in the *Cronobacter* PubMLST database (http://www.pubMLST.org/cronobacter). Strain 8706 clustered within the *Enterobacter* genus, although average nucleotide identity could not identify the species of *Enterobacter* strain 8706 (2).

Annotation using Prokka identified 4,681 coding sequences (CDSs) and 93 RNAs (6). The CDSs included those for motility, curli fimbriae, copper homeostasis, iron acquisition, multidrug efflux pumps, resistance to arsenic, magnesium, cobalt, silver, zinc, cadmium, mercury, manganese, nickel, tellurite, and lead, stress response associated with cold, osmotic, and oxidative stresses, a possible hemolysin, and several phage-associated traits. Also identified were CDSs for resistance to penicillin (including a β-lactamase), polymyxin, chloramphenicol, tetracycline, and fosmidomycin.

### Nucleotide sequence accession number.

The genome sequence of *Enterobacter* sp. strain 8706 was deposited in GenBank under the accession number LLXN00000000.
